# Spinal Cord Electrophysiology

**DOI:** 10.3791/1660

**Published:** 2010-01-18

**Authors:** Allyn Meyer, Benjamin W. Gallarda, Samuel Pfaff, William Alaynick

**Affiliations:** The Salk Institute for Biological Studies, Howard Hughes Medical Institute and Gene Expression Laboratory; Biology Graduate Program, University of California San Diego - UCSD

## Abstract

The neonatal mouse spinal cord is a model for studying the development of neural circuitries and locomotor movement. We demonstrate the spinal cord dissection and preparation of recording bath artificial cerebrospinal fluid used for locomotor studies. Once dissected, the spinal cord ventral nerve roots can be attached to a recording electrode to record the electrophysiologic signals of the central pattern generating circuitry within the lumbar cord.

**Figure:**
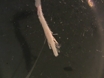


## Protocol

1. Prepare artificial cerebrospinal fluid (aCSF)1. We first prepare a 2L of a 10X stock of aCSF without magnesium or calcium. Reagents listed in millimolar. Catalog numbers refer to Sigma/Aldrich.

**Table:** 

**2 Liters 10X aCSF (without Mg or Ca)**
Reagent	mM	g/2L	Catalog
KCl	40	5.96	P-9333
NaCl	1280	149.61	S-7653
NaHCO_3_	210	35.28	S-6297
NaH_2_PO_4_	5	1.38	S-9638
Glucose	300	108.12	D-9434
Add distilled water to 2L

We will also prepare 1M solutions of MgSO_4_ and CaCl_2_ in 50mL water to allow for adjustments in molarity from experiment to experiment and to assure the calcium remains in solution.

**Table:** 

**1M Calcium and Magnesium Stocks**
Reagent	M	g/50mL	Catalog
CaCl_2_	1	7.35	C-5080
MgSO_4_	1	12.33	M-5921

The aCSF solution must be gassed with carbogen (95% O2/ 5% CO2) to lower the pH before adding calcium or the calcium will precipitate. 

**Table:** 

**1 Liter aCSF **
Reagent	Volume
10X aCSF	100mL
1M CaCl_2_	1 mL (dissecting) 2mL (recording)
1M MgSO_4_	1 mL
Add ~800 mL distilled water, gas with carbogen for 2 minutes before adding calcium
Add distilled water to 1L.

The pumps and tubing and dissecting dishes should be rinsed before and after use.

### Dissection

While dissecting, 1mM calcium aCSF should be continuously gassed with carbogen. The aCSF can be siphoned from a bottle to the dissecting dish and pumped from the dissecting dish to the bottle or sent to waste.  We use an elastomer (Sylgard, Dow Corning) coated dish to perform the dissection. 

The neonatal mouse is rapidly decapitated with sharp scissors and an incision made with scissors through the ventral thorax and abdomen. The animal is then eviscerated. The eviscerated mouse is rinsed with aCSF. The animal is then pinned to the dissecting dish with insect pins inserted through the forelimbs and at the base of the tail. 

The spinal column is removed by performing a ventral laminectomy. The spinal column is held with small forceps and one blade of small scissors is inserted immediately dorsal to the bony spinal column. The lamina is cut on one side and then the other while gently lifting the bony spinal column. This is carried out for the length of the spinal column. 

The spinal cord is removed by cutting along either side of the spinal cord to sever the spinal nerve roots and cut the meninges surrounding the spinal cord. We cut on the left and right sides, then we cut the meninges attached to the dorsal side of the spinal cord to free it. The isolated cord is then pinned to the dish near the aCSF inlet to assure good oxygenation if additional animals are dissected. 

The isolated spinal cord is then transferred to a recording dish using a small spoon or spatula. Here we perfuse the preparation with oxygenated 2mM calcium aCSF. The spinal cord is pinned to the elastomer coated dish and micromanipulators used to move extracellular, whole root suction electrodes near the roots. When the electrode tip is near the free root end, gentle suction is applied to draw the root into the suction electrode. This process may be repeated to record several roots simultaneously.


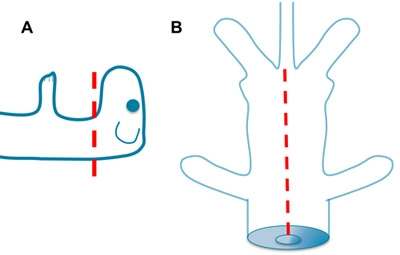
 **Figure 1. Decapitation and Evisceration.** A. Rapidly decapitate the mouse with sharp scissors. Greater or lesser contributions from the brain stem can be achieved depending on the level of cut. B. Place one blade of the scissors into the opening of the thoracic cavity created by the decapitation. Open the ventral thorax and abdomen to the base of the tail. Remove the viscera and rinse with aCSF. 


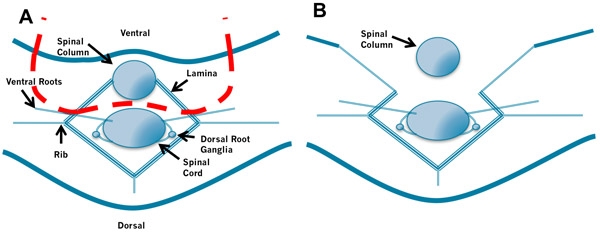
 **Figure 2. Removal of the Spinal Column.** A. Insert the left blade of the scissors into the space between the spinal column and spinal cord to the right of the spinal cord and cut through the bones laying ventral to the cord. Then insert the right blade of the scissors into the space between the spinal column and spinal cord to the left of the spinal cord and cut. B. Repeat this process while applying gentle traction to the spinal column. Take care not to puncture the spinal cord by holding the scissors at a low angle. 


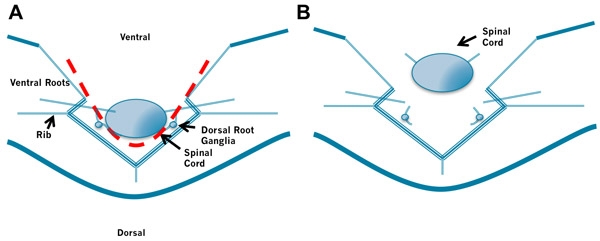
 **Figure 3. Removal of the Spinal Cord.** A. Insert the left blade of the scissors into the space between the spinal cord and the bones to the right of the spinal cord and cut through the spinal roots and meninges laying lateral to the cord. Then insert the right blade of the scissors into the space between the spinal cord and the bones to the left of the spinal cord and cut through the spinal roots and meninges laying lateral to the cord. B. Finally, gently lift the rostral cord and cut the meninges connecting the dorsal cord to the laminae.  Again take care not to puncture the spinal cord by holding the scissors at a low angle. Do not cut the roots too close to the spinal cord.

## Discussion

The isolated neonatal spinal cord provides a tractable method of studying nervous system development1,2. Within the lumbar spinal cord of neonatal rodents, central pattern generating circuitry can produce fictive locomotion in the presence of neurotransmitters. This fictive locomotion consists of rhythmic increases in activity, bursts, that are produced at 0.2 to 0.5 Hz. These bursts are organized to produce left-right alternation along the length of the cord and flexor-extensor alternation (ipsilateral L2 relative to L5). Several genetic mutations in mice have been shown to have abnormal behavior 3-5. Understanding how the neural circuitry of the spinal cord is altered during development and genetic perturbations will inform studies of neural circuitry elsewhere in the CNS. 

It is notable that there are several standard aCSF solutions in regular use. Below is a table of commonly used aCSF preparations.

### Table 1: Common aCSF Compositions

**Table:** 

**Laboratory**	**aCSF Composition in mM**
Pfaff, O’Donovan, Landmesser (3)	128 NaCl, 4 KCl, 1.5 CaCl_2_, 1 MgSO_4_, 0.5 NaH_2_PO_4_, 21 NaHCO_3_, 30 glucose.
Goulding, Keihn (5)	111 NaCl, 3.08 KCl, 2.52 CaCl_2_, 1.25 MgSO_4_, 1.18 KH_2_PO_4_, 25 NaHCO_3_, 11 glucose
Brownstone (6)	127 NaCl, 1.9-3.9 KCl, 1.2 KH_2_PO_4_, 2.4 CaCl_2_, 1.3 MgCl_2_, 26 NaHCO_3_,10 glucose
Ziskind-Conhaim Dissection (7)	113 NaCl, 3 KCl, 1 CaCl_2_, 6 MgCl_2_, 25 NaHCO_3_, 1 NaH_2_PO_4_, 11 glucose
Ziskind-Conhaim Extracellular (7)	113 NaCl, 3 KCl, 2 CaCl_2_, 1 MgCl_2_, 25 NaHCO_3_, 1 NaH_2_PO_4_, 11 glucose.
O’Donovan, chick (8,9)	139 NaCl, 2.9-5 KCl, 17 NaHCO_3_, 1 MgCl_2_, 3 CaCl_2_, 12 glucose
